# *In situ* solid-state fabrication of hybrid AgCl/AgI/AgIO_3_ with improved UV-to-visible photocatalytic performance

**DOI:** 10.1038/s41598-017-12625-8

**Published:** 2017-09-28

**Authors:** Jing Xie, Yali Cao, Dianzeng Jia, Yizhao Li, Kun Wang, Hui Xu

**Affiliations:** Key Laboratory of Energy Materials Chemistry, Ministry of Education, Key Laboratory of Advanced Functional Materials, Autonomous Region, Institute of Applied Chemistry, Xinjiang University, Urumqi, 830046 Xinjiang P.R. China

## Abstract

The AgCl/AgI/AgIO_3_ composites were synthesized through a one-pot room-temperature *in situ* solid-state approach with the feature of convenient and eco-friendly. The as-prepared composites exhibit superior photocatalytic performance than pure AgIO_3_ for the degradation of methyl orange (MO) under both UV and visible light irradiation. The photodegradation rate toward MO of the AgCl/AgI/AgIO_3_ photocatalyst can reach 100% after 12 min irradiation under UV light, or 85.4% after 50 min irradiation under visible light, being significantly higher than AgCl, AgI, AgIO_3_ and AgI/AgIO_3_. In addition, the AgCl/AgI/AgIO_3_ photocatalyst possesses strong photooxidation ability for the degradation of rhodamine B (RhB), methylene blue (MB), phenol, bisphenol A (BPA) and tetracycline hydrochloride under visible light irradiation. The reactive species capture experiments confirmed that the h^+^ and •O^2−^ play an essential role during the photocatalytic process under UV light or visible light irradiation. The enhanced effect may be beneficial from the enhanced light adsorption in full spectrum and increased separation efficiency of photogenerated hole-electron pairs, which can be ascribed to the synergistic effect among AgCl, AgI and AgIO_3_ nanoplates in AgCl/AgI/AgIO_3_ composites.

## Introduction

Semiconductor photocatalytic oxidation technique has attracted widespread attention on the degradation of environmentally hazardous substances due to its potential utilization of solar energy, the high conversion rate of solar energy and the strong oxidation ability^1,2^. In recent years, nanoscaled TiO_2_ have been regarded as one of most promising materials in photocatalytic field because of its high photocatalytic activity, stability and non-poisonous^3–5^. Nevertheless, TiO_2_ has a wide band gap of 3.2 eV which lead to low quantum yields as well as the lack utilization of visible light (the main component of solar spectrum)^6,7^. Therefore, photocatalyst with full-spectrum catalysis ability are highly desired for environmental purification.

Recently, a growing number of Ag-based oxy-acid salts photocatalysts, such as Ag_3_PO_4_^8^, Ag_3_VO_4_^9^, Ag_2_WO_4_^10^, Ag_2_CO_3_^11^ and AgIO_3_^12^ have exhibited novel catalysis efficiency on the degradation of environmental hazardous. Among them, AgIO_3_ has aroused a great deal attention in photocatalytic field due to the lone pair electrons of I^5+^ is beneficial to form layered structure in crystal^13^. Unfortunately, just like TiO_2_, the band gap of AgIO_3_ was empirically calculated to be 3.38 eV, leading to its sluggish reaction to visible light^14^. To date, composite photocatalysts have been proved to be a feasible strategy to overcome the drawbacks via the so-called synergetic effects. To optimize the photocatalytic performance of AgIO_3_, researchers have attempted to couple AgIO_3_ with other Ag-based materials to upgrade its response to visible light and inhibit the recombination of electron-holes^15,16^. For instance, He *et al*. has reported that Ag/AgIO_3_ composites were fabricated by the combination of the solid-state and liquid phase method, it displayed high photocatalytic activity in the conversion of CO_2_ to CH_4_ and CO under visible light irradiation^17^. Zeng *et al*. prepared Ag/AgI/AgIO_3_ by a hydrolysis method and followed an *in-situ* reduction reaction, the composites also exhibited better photocatalytic performance than pure AgIO_3_ under both UV light and visible light irradiation^18^. Despite a few successful examples have convinced that composite structures can improve the photocatalytic activity of pure AgIO_3_, the multiple-steps operations for preparing Ag-based composites continuously limit their large scale application due to the complicated procedures that caused time and energy consumption. Thus, it is necessary to develop a simple and efficient approach for rationally fabricating Ag-based composites.

Herein, the AgCl/AgI/AgIO_3_ composites was designed and synthesized by a one-pot solid-state technique utilizing AgIO_3_ as a self-sacrificing template and HONH_3_Cl as reducing agent. This method has the feature of simplicity, rapidity, low cost and high yield^19–21^. The photocatalytic performance of the AgCl/AgI/AgIO_3_ composites was evaluated by the decomposition of various kinds of organic contaminants under visible light irradiation. Furthermore, the photocatalytic mechanisms of the AgCl/AgI/AgIO_3_ composites correlating to different light source were also systematically investigated. This study may open up a promising approach to design and synthesize composite photocatalysts for environmental purification.

## Results and Discussions

**Figure 1:**
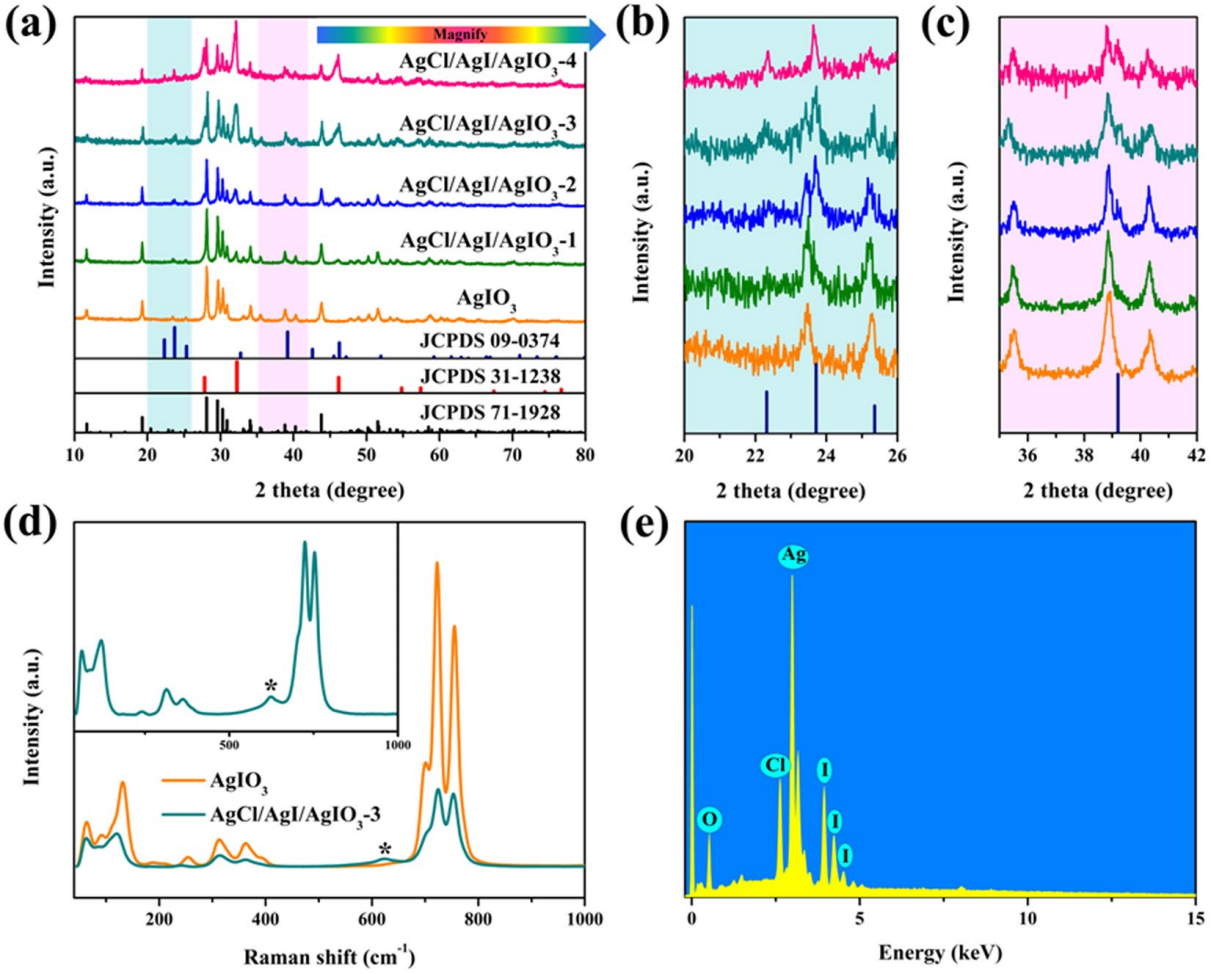
XRD patterns (**a**–**c**) and Raman spectrum (**d**) of the pure AgIO_3_ and AgCl/AgI/AgIO_3_ composites, (**e**) EDS of the AgCl/AgI/AgIO_3_ composites.

Figure 1a showed the XRD patterns of the as-prepared pure AgIO_3_ and AgCl/AgI/AgIO_3_ composites. As demonstrated in Fig. 1a, the diffraction peaks of pure AgIO_3_ at 2θ values of 11.60°, 19.29°, 28.09°, 29.65°, 30.30°, 30.91°, 34.11°, 38.87°, 43.86° and 51.5° were assigned to (020), (021), (041), (211), (230), (002), (231), (001), (232) and (271) crystal planes of the orthorhombic AgIO_3_ (JCPDS 71-1928), respectively. No impurity peaks were found, indicating the high purity of the pure AgIO_3_. In AgCl/AgI/AgIO_3_ composites, peaks located at 2θ = 27.80°, 32.16° and 46.18° were appeared, which match well with (111), (200) and (220) planes of cubic AgCl (JCPDS 31-1238), respectively. Besides, as observed in the enlarged XRD patterns (Fig. 1b and c), two small peaks situated at 2θ values of 23.70° and 39.20° were attributed to (002) and (110) planes of AgI (JCPDS 09-0374), respectively. Furthermore, with the increase of the amount of reductant, the characteristic peaks of AgIO_3_ decreased in intensity gradually, while the characteristic peaks of AgCl and AgI increased in intensity simultaneously, which verifies the conversion from AgIO_3_ to AgCl and AgI through the solid-state reaction between AgIO_3_ and HONH_3_Cl. However, the position of the diffraction peaks belong to AgIO_3_ did not shift in AgCl/AgI/AgIO_3_ composites, which suggested that the crystal phase structure of AgIO_3_ did not change after the introducing of AgCl and AgI. Moreover, the Raman spectrum of the pure AgIO_3_ and AgCl/AgI/AgIO_3_-3 (Fig. 1d) were tested, it was clearly seen that the Raman bands of the samples are almost the same, whereas the intensity of AgCl/AgI/AgIO_3_-3 is significantly decreased compared to pure AgIO_3_, which further illustrated that the *in situ* solid-state reaction occurred between AgIO_3_ and HONH_3_Cl. A new characteristic peak appears at 623.5 cm^−1^, which can be ascribed to the vibration peak of AgCl^22,23^, the result is coincident with the result of XRD. To further identify the component elements of AgCl/AgI/AgIO_3_ composites, the EDS of the AgCl/AgI/AgIO_3_-3 were performed. As depicted in Fig. 1e, it also confirmed the coexistence of Ag, O, Cl and I in the AgCl/AgI/AgIO_3_-3 composites.

**Figure 2:**
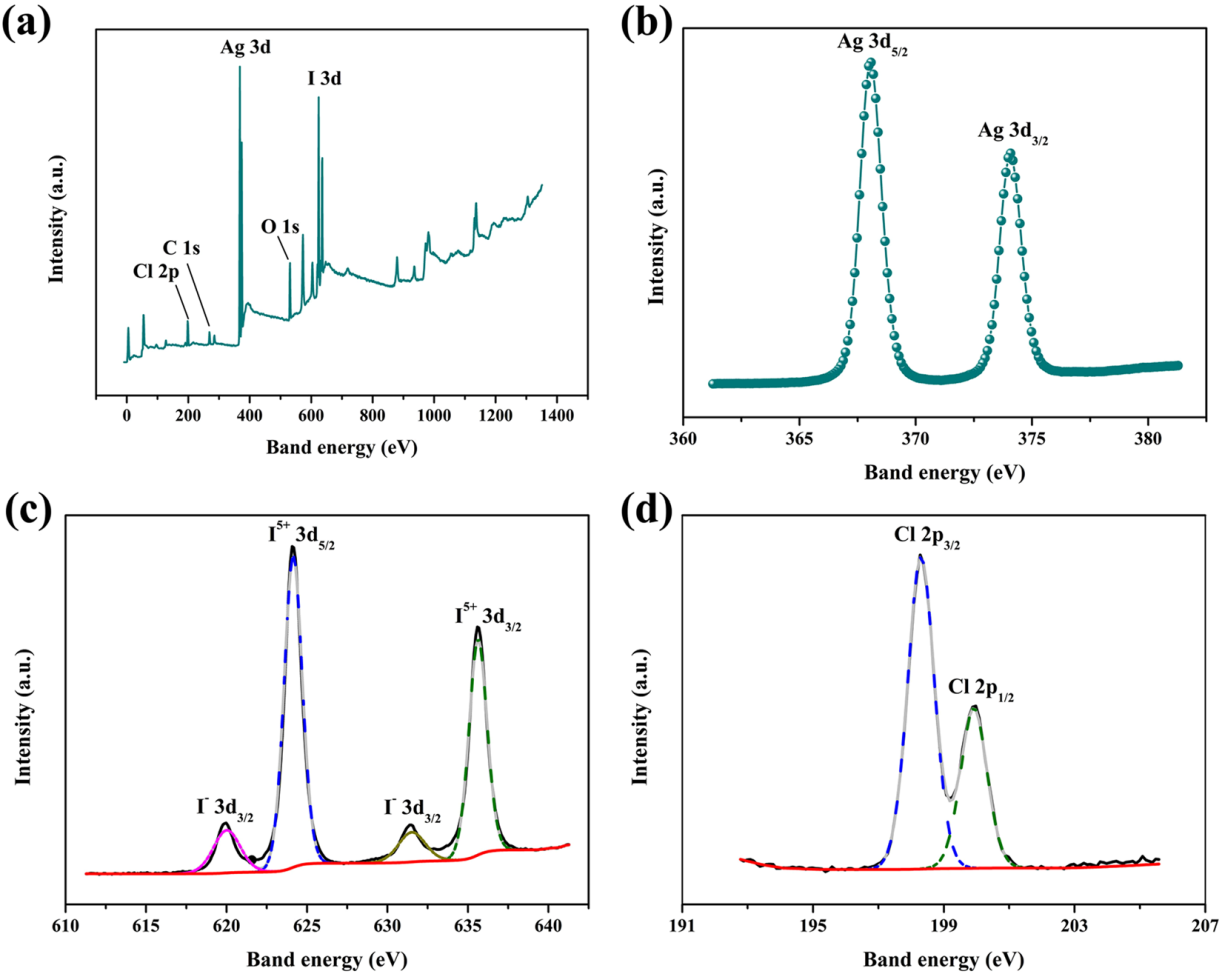
XPS spectra of the AgCl/AgI/AgIO_3_-3: (**a**) survey scan, (**b**) Ag 3d, (**c**) I 3d, (**d**) Cl 2p.

To provide direct evidence for confirming the surface chemical composition and the chemical states of the as-prepared samples, the XPS full-scan spectrum was employed. Wherein only the Ag 3d, O 1 s, I 3d, Cl 2p and a trace of C 1 s species were detected (Fig. 2a). The corresponding high-resolution XPS spectra in Fig. 2b–d display the characteristic peaks of Ag 3d, I 3d and Cl 2p over the AgCl/AgI/AgIO_3_-3 sample. Peaks with binding energies of 368.08 eV and 374.08 eV are associated with Ag^+^ 3d_5/2_ and Ag^+^ 3d_3/2_ peaks, respectively (Fig. 2b). Two peaks at 624.08 eV and 635.58 eV can be ascribed to I^5+^ 3d_5/2_ and I^5+^ 3d_3/2_, and meantime the peaks at 619.98 eV and 631.48 eV match the features of I^−^ 3d_5/2_ and I^−^ 3d_3/2_, respectively (Fig. 2c). This result demonstrates the co-existence of I^5+^ and I^−^. As shown in Fig. 2d, the peaks at 198.28 and 199.98 eV in AgCl/AgI/AgIO_3_-3 are detected, which can be ascribed to Cl 2p_3/2_ and Cl 2p_1/2_. The XPS results confirmed the three substances (AgCl, AgI and AgIO_3_) exist simultaneously in the AgCl/AgI/AgIO_3_-3 sample, further illustrating the formation of the composites.

**Figure 3:**
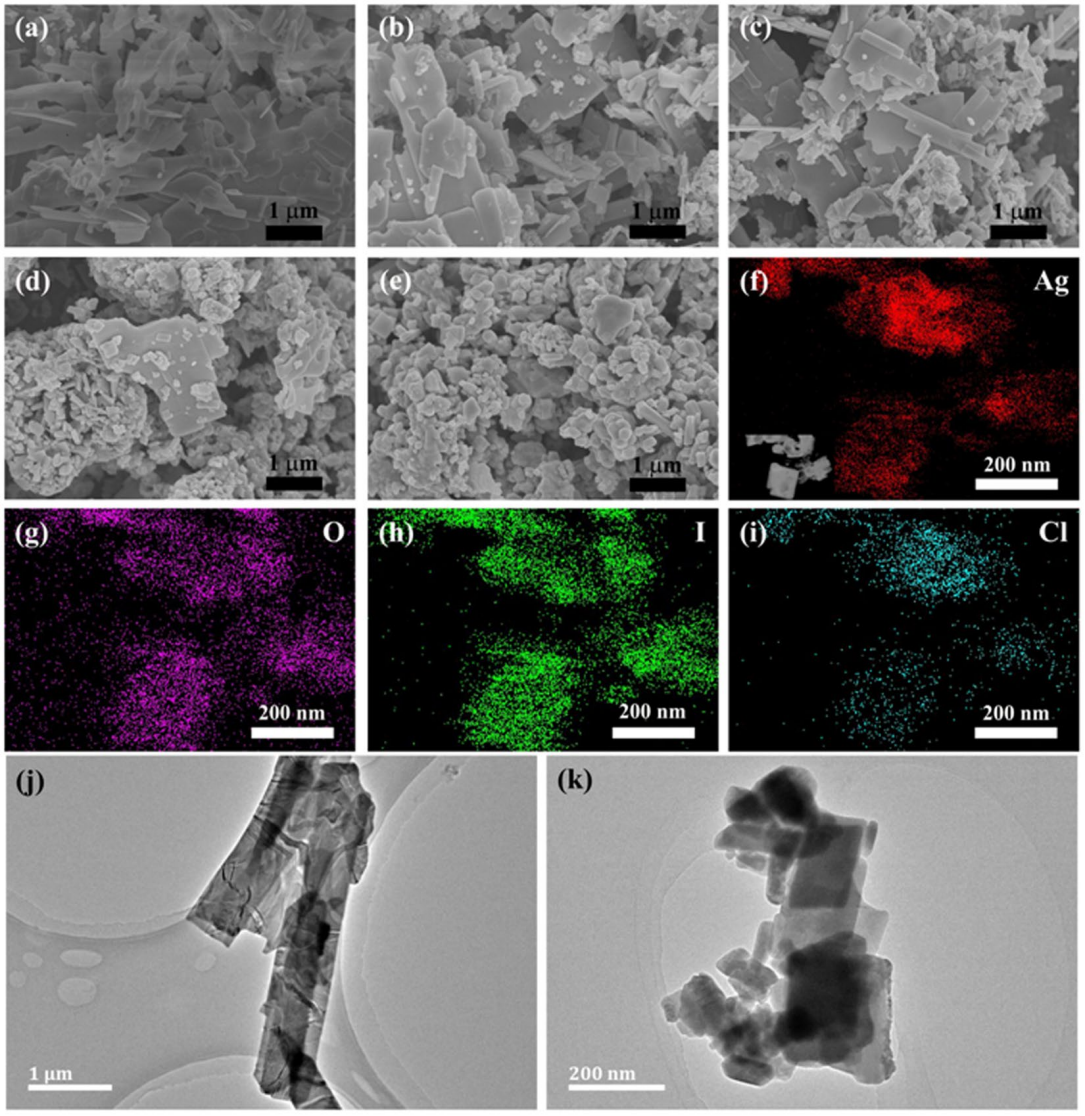
FESEM images of pure AgIO_3_ (**a**), AgCl/AgI/AgIO_3_-1 (**b**), AgCl/AgI/AgIO_3_-2 (**c**), AgCl/AgI/AgIO_3_-3 (**d**), and AgCl/AgI/AgIO_3_-4 (**e**), respectively. EDS mapping of AgCl/AgI/AgIO_3_-3 (**f**–**i**). TEM images of pure AgIO_3_ (**j**) and AgCl/AgI/AgIO_3_-3 (**k**), respectively.

To investigate the morphology evolution of various samples, the FESEM imaging was measured. As displayed in Fig. 3a, the pure AgIO_3_ is major composed of nanosheets with smooth surfaces, and the sizes of these nanosheets are approximately 2~4 μm × 500~600 nm (length × wide). However, the sizes of the nanosheets become smaller and a certain amount of irregular nanoparticles are distributed on the surface and vicinity of AgIO_3_ nanosheets after the introduction of the reducing agent, illustrating that the formation of AgCl and AgI. With increasing amount of HONH_3_Cl, the sizes of the AgIO_3_ nanosheets become smaller, meanwhile, more AgCl and AgI nanoparticles generate. Figure 3(b–e) clearly shows the morphology evolutions from AgIO_3_ nanosheets to Ag-AgI-AgIO_3_ composites with irregular nanoparticles. EDS mapping was employed to further identify the elemental composition and distribution of pure AgIO_3_ and AgCl/AgI/AgIO_3_-3. Supplementary Fig. S1 reveals that Ag, O and I elements exist in pure AgIO_3_ and all of them were uniformly distributed in AgIO_3_. After reduced by HONH_3_Cl, Cl element was introduced into this system and with the other three elements (Ag, O and I) well-distributed in AgCl/AgI/AgIO_3_-3 (Fig. 3f–i), once again illustrating that the AgCl and AgI was well distributed on the AgIO_3_.

TEM was also employed to investigate the morphology of the pure AgIO_3_ and AgCl/AgI/AgIO_3_-3. As shown in Fig. 3j, pure AgIO_3_ was composed of regular rectangular nanosheets with 4 μm in length and 600 nm in width. However, the shape of the perfect nanosheets was destroyed after the accretion of reductant, as displayed in Fig. 3k. The sizes of the nanosheets decreased, in addition, there are a certain amount of substances with irregular morphologies appeared on the surface or the edge of the nanosheets. This phenomenon also can be observed in others regions of the sample, as seen in the low-magnification TEM (Supplementary Fig. S2), which is consistent with the SEM result.

**Figure 4:**
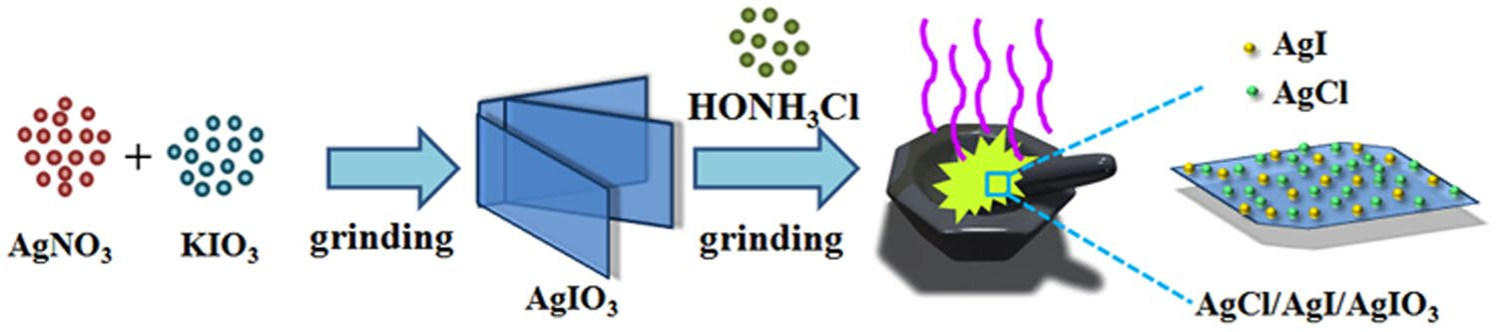
Schematic illustration of the possible formation process of the AgCl/AgI/AgIO_3_ composites.

To the best of our knowledge, composite structures are generally obtained through several multiple-step approaches that include *in situ* reduction reaction, photoreduction and ion-exchange reaction in solutions^24–26^. Although, these methods can obtain the materials with tailored properties by adjusting the reactive condition in each step, they are addicted to complicated process and time consumption. In this study, a simple and rapid one-pot *in situ* solid-state method was first applied to fabricate AgCl/AgI/AgIO_3_ composites and the AgIO_3_ nanosheets was employed as a self-sacrificing template. A schematic illustration of the possible formation process of the AgCl/AgI/AgIO_3_ composites was shown in Fig. 4. Firstly, the AgIO_3_ nanoplates were obtained by a metathesis reaction between AgNO_3_ and KIO_3_ powder at room temperature. The process accompanied with the release of heat and vapor, the moist powders were acquired after grinding 10 min and the AgIO_3_ nanosheets produced. Subsequently, the prepared AgIO_3_ was reacted with HONH_3_Cl by an *in situ* oxidation-reduction reaction in solid state. Interesting, the HONH_3_Cl plays a dual role in this process, which is not only as a reducing agent, but also provides the chloride ion. When the HONH_3_Cl added into this reaction system, the purple gas comes out at once and the color of the product changes from white to yellow-green as soon as possible. Based on the above phenomena, the reaction that occurred in the formation of AgCl/AgI/AgIO_3_ crystal nuclei may be explained by equations (1 and 2):

1 $${{\rm{AgNO}}}_{3}+{{\rm{KIO}}}_{3}={{\rm{AgIO}}}_{3}+{{\rm{KNO}}}_{3}$$

2 $$\begin{array}{rcl}16{{\rm{AgIO}}}_{3}+12{{\rm{HONH}}}_{3}{\rm{Cl}} & = & 2{{\rm{AgIO}}}_{3}+12{\rm{AgCl}}+2{\rm{AgI}}+6{{\rm{I}}}_{2}\uparrow +6{{\rm{N}}}_{2}\uparrow \\  &  & +15{{\rm{O}}}_{2}\uparrow +24{{\rm{H}}}_{2}{\rm{O}}\end{array}$$

**Figure 5:**
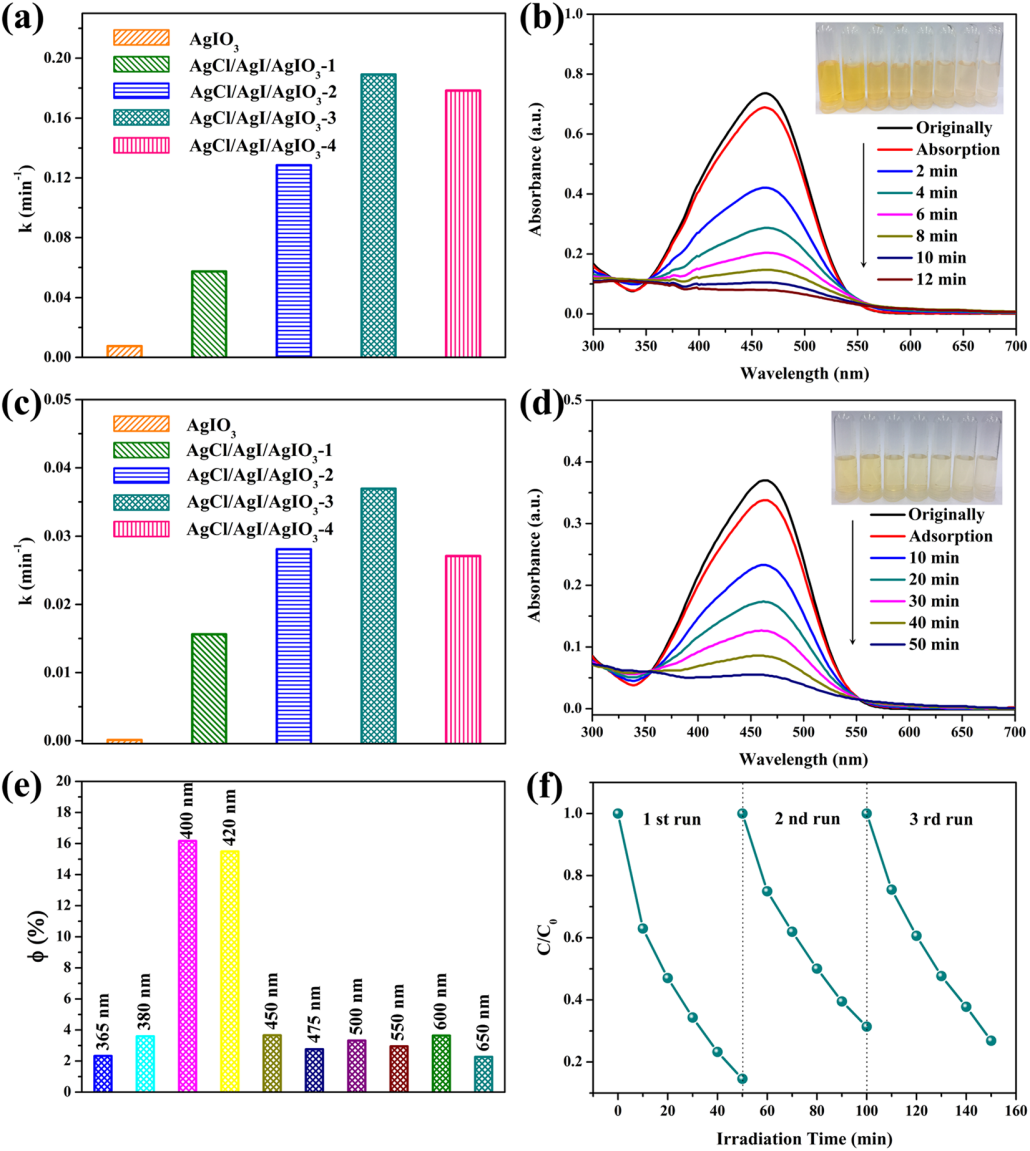
(**a**) The apparent first-order rate constants of photocatalytic degradation of MO over AgIO_3_ and AgCl/AgI/AgIO_3_ composites under UV light irradiation, (**b**) temporal UV-vis spectral change of MO with irradiation time over AgCl/AgI/AgIO_3_-3 under UV light irradiation, (**c**) the apparent first-order rate constants of photocatalytic degradation of MO over AgIO_3_ and AgCl/AgI/AgIO_3_ composites under visible light irradiation, (**d**) temporal UV-vis spectral change of MO with irradiation time over AgCl/AgI/AgIO_3_-3 under visible light irradiation, (**e**) the calculated apparent quantum yields over the AgCl/AgI/AgIO_3_-3 composite depended on wavelength, (**f**) recycling experiments were conducted on the visible photocatalytic degradation of MO by AgCl/AgI/AgIO_3_-3 composites.

Since AgIO_3_ is a typical photocatalyst that only responded in the ultraviolet region, the photocatalytic activities of pure AgIO_3_ and AgCl/AgI/AgIO_3_ composites were first investigated through degrading MO aqueous solution under UV light irradiation. As displayed in Fig. 5a, all the AgCl/AgI/AgIO_3_ composites possess better photocatalytic activity on the degradation of MO under UV light irradiation than pure AgIO_3_. The apparent first-order rate constants of the as-prepared photocatalysts first increased gradually and then decreased along with increase in the amount of HONH_3_Cl. The AgCl/AgI/AgIO_3_-3 composites exhibited the best photocatalytic activity in all photocatalysts and it’s apparent first-order rate constants is 18.91 × 10^−2^ min^−1^, which is about 24.8 times of pure AgIO_3_ (0.76 × 10^−2^ min^−1^). In Fig. 5b, the typically absorption peak of AgCl/AgI/AgIO_3_-3 photocatalyst towards the MO is decreased dramatically with the increase of illumination time and the color of the dye become faded, until disappeared and colourless. The degradation of MO over the AgCl/AgI/AgIO_3_-3 is 100% with the irradiation of UV light after 12 min. Since the sun light is mainly composed of ~5% ultraviolet light and 43% visible light, the exploration of photocatalysts which can make full use of sunlight is the primary task in this field^27,28^. Accordingly, the photocatalytic performances of the as-prepared photocatalysts were also investigated under visible light excitation. As seen in Fig. 5c, the trend of the photocatalytic oxidation of MO over all photocatalysts is basically consistent with the results with UV irradiation. The AgCl/AgI/AgIO_3_-3 composites still shows the outstanding photooxidation ability towards MO and it’s photodegradation rate constants is 3.69 × 10^−2^ min^−1^, which is much higher than the pure AgIO_3_ (0.01 × 10^−2^ min^−1^) and other AgCl/AgI/AgIO_3_ composites as well. In Fig. 5d, the typically absorption peak of the MO over AgCl/AgI/AgIO_3_-3 photocatalyst is also diminished rapidly and the color of the dye also become shallower as the increase of illumination time. It is calculated that the degradation of MO over the AgCl/AgI/AgIO_3_-3 is 85.4% after 50 min irradiation of visible light. The photocatalytic activity of AgCl/AgI/AgIO_3_-3 was better than the pure AgCl, AgI and AgI/AgIO_3_ composite, as presented in Supplementary Figs S3 and S4, illustrating the important role of synergistic effect in AgCl/AgI/AgIO_3_-3. In addition, the AgCl/AgI/AgIO_3_-3 composite still reveals a better performance on the photocatalytic degradation of MO under both UV and visible light irradiation than the mechanical mixtures of AgCl, AgI and AgIO_3_ (see Supplementary Fig. S5), which further indicated the importance of the *in situ* reaction to the synergistic effects.

In order to investigate the best excitation wavelength and the light absorption properties of AgCl/AgI/AgIO_3_-3 composite, the wavelength dependent photocatalytic activity of AgCl/AgI/AgIO_3_-3 was tested for the degradation of MO by a 300 W Xe with different band-pass filter (Supplementary Fig. S6). Besides, the wavelength dependent photocatalytic activities over AgCl/AgI/AgIO_3_-3 were also calculated as apparent quantum yields^29,30^, as displayed in Fig. 5e. The AgCl/AgI/AgIO_3_-3 composite features excellent photooxidation ability for MO under all test wavelengths and shows high photocatalytic activity at 400 nm, suggesting that AgCl/AgI/AgIO_3_-3 composites has a good photoresponse to the full-spectrum and also implying that 400 nm was the best excitation wavelength. In order to evaluate the repeatability and stability of the photocatalyst, the recycling experiment was conducted on the visible photocatalytic degradation of MO. Figure 5f shows that there is a little decrease after three consecutive cycles, which could attribute to the conversion of a part of I^5+^ to I^−^ (Supplementary Fig. S7). The reaction changed the relative contents of AgCl, AgI and AgIO_3_, which is the disadvantage for recycling.

**Figure 6:**
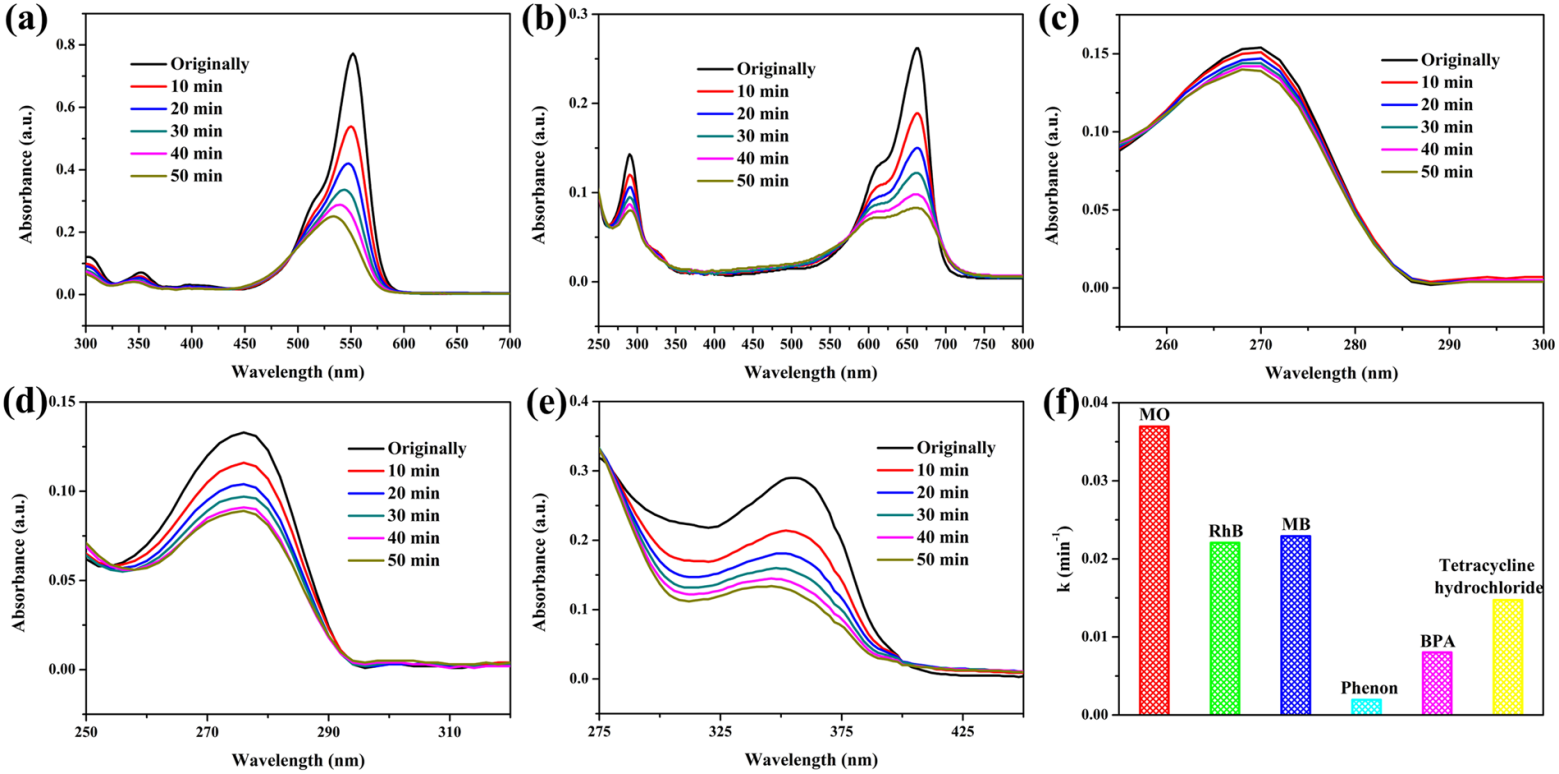
Temporal UV-vis spectral change of RhB (**a**), MB (**b**), phenol (**c**), BPA (**d**) and tetracycline hydrochloride (**e**) with irradiation time over AgCl/AgI/AgIO_3_-3 composite, (**f**) the apparent first-order rate constants of photocatalytic degradation of MO, RhB, MB, phenol, BPA and tetracycline hydrochloride over AgCl/AgI/AgIO_3_-3 composite under visible light irradiation.

Different kinds of organic pollutants had been used to evaluate the photocatalytic performance of the AgCl/AgI/AgIO_3_-3 composite comprehensively, besides cationic dye MO, anionic dye rhodamine B (RhB) and methylene blue (MB), neutral stubborn pollutants phenol, bisphenol A (BPA) and tetracycline hydrochloride. Figure 6a–e displays the typically absorption peak of RhB, MB, phenol, BPA and tetracycline hydrochloride, respectively, over AgCl/AgI/AgIO_3_-3 photocatalyst with visible light irradiation. Surprisingly, the AgCl/AgI/AgIO_3_-3 photocatalyst possesses the excellent degradation effects on all types of pollutants except for phenol. The low degrading efficiency of phenol may due to the P-π conjugative effect, which lead to the fracture of C-O bond is very difficult (Supplementary Fig. S9). The apparent first-order rate constants for the degradation of MO, RhB, MB, phenol, BPA and tetracycline hydrochloride over AgCl/AgI/AgIO_3_-3 photocatalyst is 3.69 × 10^−2^ min^−1^, 2.21 × 10^−2^ min^−1^, 2.29 × 10^−2^ min^−1^, 0.20 × 10^−2^ min^−1^, 0.80 × 10^−2^ min^−1^ and 1.47 × 10^−2^ min^−1^, respectively (Fig. 6f), illustrating the AgCl/AgI/AgIO_3_-3 photocatalyst almost can degrade various kinds of organic pollutants effectively.

**Figure 7:**
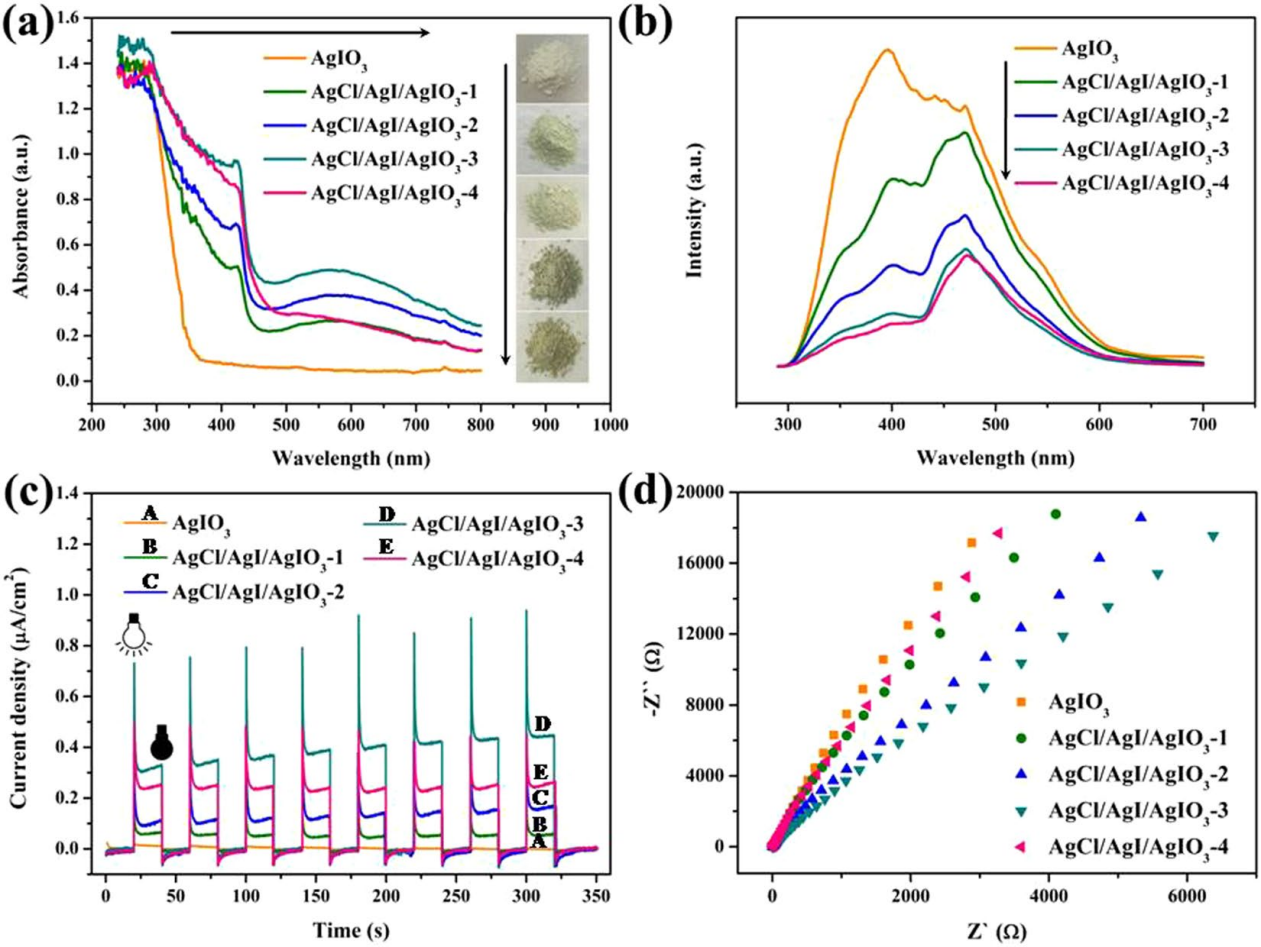
UV-vis diffuse reflectance spectroscopy and photograph (**a**), fluorescence spectrum with 280 nm excitation wavelength (**b**), photocurrent density with visible light irradiation (**c**) and EIS Nyquist plots (**d**) of pure AgIO_3_ and AgCl/AgI/AgIO_3_ composites.

Composites are always considered as effective means to improve photocatalytic activity owing to the tunable band structures and efficient electron-hole separation and transportation^31,32^. The UV-vis diffuse reflectance spectroscopy was employed to study the light absorbance property of the as-synthesized samples. As revealed in Fig. 7a, the absorption threshold of pure AgIO_3_ is situated at about 350 nm and the band gap energy of pure AgIO_3_ (3.8 eV) was calculated through the Tauc plot extrapolation (see Supplementary Fig. S10), indicating that the pure AgIO_3_ is unresponsive to visible light. While the as-prepared AgCl/AgI/AgIO_3_ composites exhibit enhanced adsorption in full spectrum with the increase amount of HONH_3_Cl, especially the absorption range at 300–420 nm and 450–800 nm, which should be assigned to the generation of AgCl and AgI on the surface of AgIO_3_. Particularly, the AgCl/AgI/AgIO_3_-3 sample exhibits the supreme absorption among all the samples, implying the best photocatalytic activity. In addition, the color of the as-prepared samples changes from white to light-green, and finally to yellow-green (the insert of Fig. 7a), suggesting that the orderly enhanced response to visible light with the increase in the amount of HONH_3_Cl. Based on the above analysis, the light absorption can be effectively strengthened by the collaborative effects of AgCl, AgI and AgIO_3_.

Subsequently, the photoluminescence (PL) spectrum was employed to appraise the charge separation of electron-hole pairs^33^. As shown in Fig. 7b, all the AgCl/AgI/AgIO_3_ composites demonstrate significant decrease of the photoluminescence emission intensity as compared to pure AgIO_3_, and the intensity gradually decreased with the increase of HONH_3_Cl, illustrating that the AgCl/AgI/AgIO_3_ composites can effectively hinder the recombination of electron-hole pairs and the amount of HONH_3_Cl can optimize the phtocatalytic ativity of AgIO_3_. The AgCl/AgI/AgIO_3_-3 sample also reveals the lowest intensity of photoluminescence emission, suggesting that AgCl/AgI/AgIO_3_-3 has the best electron-hole separation property. Time resolved photoluminescence spectra of pure AgIO_3_ and AgCl/AgI/AgIO_3_-3 composite also indicated more non-radiative transfer of photogenerated charge carriers in AgCl/AgI/AgIO_3_-3 composite (Supplementary Fig. S11).

The photocurrents density of photocatalyst can further auxiliarily illustrate the separation and transfer of photo-generated electrons and holes^34^. As displayed in Fig. 7c, the photocurrents densities of pure AgIO_3_ and the AgCl/AgI/AgIO_3_ were investigated under visible light irradiation and all of them were reversible and stable when the light on or light off. The photocurrents density of AgCl/AgI/AgIO_3_ composites firstly increased gradually and then decreased with the increase of HONH_3_Cl. Additionally, the AgCl/AgI/AgIO_3_-3 composite possesses the highest photocurrents density, reflecting its optimal photocatalytic activity, which is consistent with the PL analysis.

The electrochemical impedance spectroscopy (EIS) responses of the samples were also used to monitor the charge migration process on the electrodes, as presented in Fig. 7d. Customarily, the smallest arc radius on the EIS Nyquist plot indicated efficient separation of the photogenerated electrons and holes^35^. The arc radius of AgCl/AgI/AgIO_3_-3 is smaller than that of pure AgIO_3_ and other AgCl/AgI/AgIO_3_, suggesting the improved transfer efficiency of photogenerated carriers in AgCl/AgI/AgIO_3_-3 composites. On the basis of the above analysis, the visible light absorption properties, the separation and transfer of photo-generated electrons and holes can be effectively enhanced by the formation of AgCl/AgI/AgIO_3_ composites, thus achieving superior photocatalytic performance.

**Figure 8:**
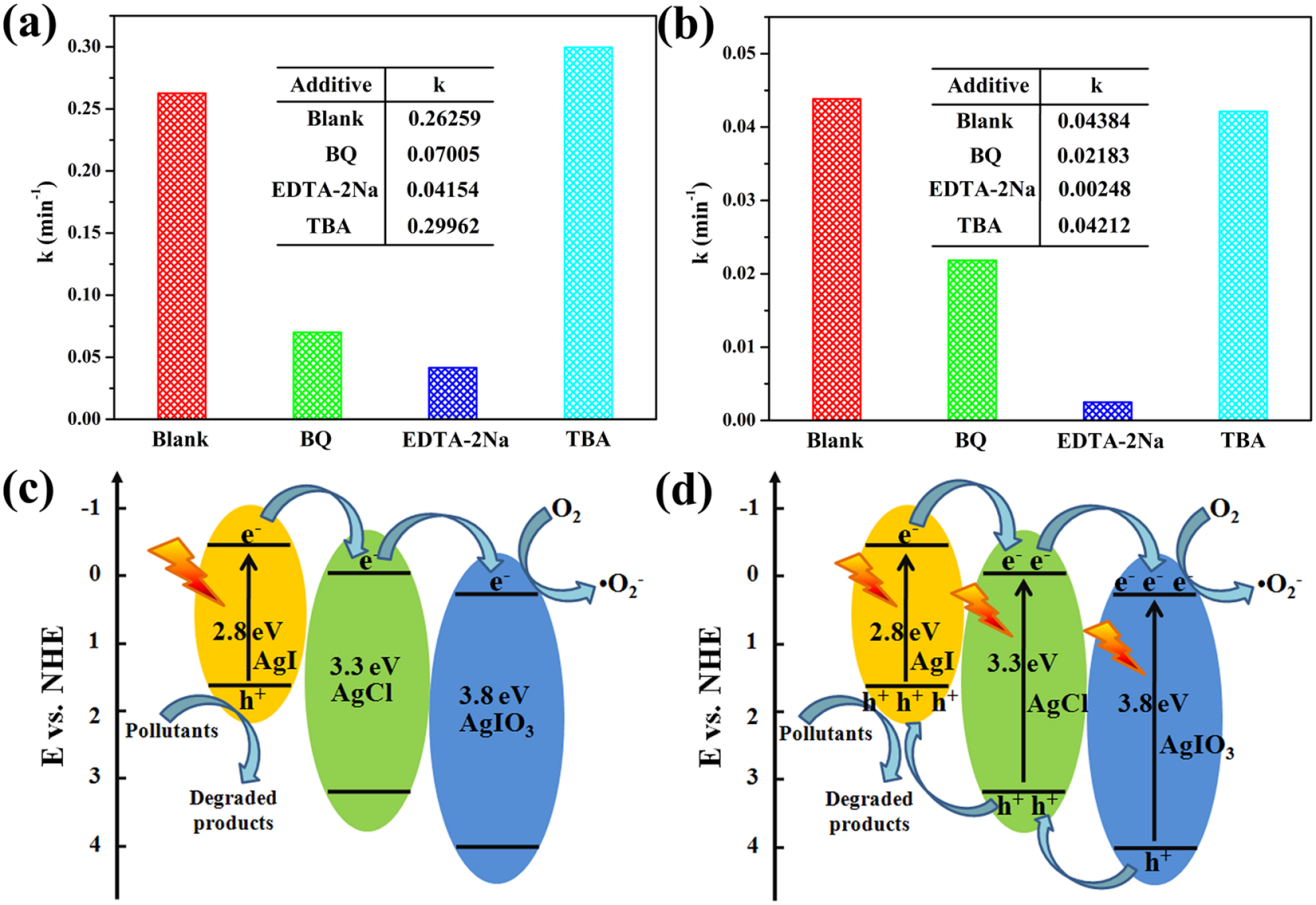
The plots of photogenerated charges trapping experiment on the photocatalytic degradation of MO over AgCl/AgI/AgIO_3_-3 composites under UV light (**a**) or visible light irradiation (**b**), photocatalytic mechanism process of AgCl/AgI/AgIO_3_-3 under under UV light (**c**) or visible light irradiation (**d**).

To distinguish the role of active radicals on the photocatalytic degradation of MO over AgCl/AgI/AgIO_3_-3 based on different light source, the trapping experiment was conducted. As revealed in Fig. 8a and b, ethylene diamine tetraacetic acid disodium salt (EDTA-2Na), benzoquinone (BQ) and tertiary butanol (TBA) were selected as scavengers for h^+^, •O^2−^ and •OH, respectively. When the EDTA-2Na was added, the photocatalytic performance of AgCl/AgI/AgIO_3_-3 under both UV light and visible light was inhibited greatly, which illustrated that h^+^ was the primary active species. The photocatalytic oxidation ability of AgCl/AgI/AgIO_3_-3 was also restrained by the addition of BQ, indicating that •O^2−^ played an auxiliary role on the photocatalytic degradation of MO over AgCl/AgI/AgIO_3_-3 under different light source. The apparent rate constant k didn’t change after the TBA added, which suggested that •OH was not affecting the photocatalytic performance of AgCl/AgI/AgIO_3_-3.

Based on the abovementioned experimental results, the possible photocatalytic mechanism process was proposed in Fig. 8. When the photocatalysis reaction system with AgCl/AgI/AgIO_3_-3 exposed to UV light (Fig. 8c), AgCl, AgI and AgIO_3_ all can be activated by UV light photons and accompany electrons jumping from the valence band (VB) to the conduction band (CB). As depicted in Supplementary Table S1, the calculated CB energy levels of AgIO_3_ is 0.26 eV, which is more positive than the CB potentials of AgI (−0.42 eV) and AgCl (−0.06 eV). Therefore, the photoinduced electrons of AgI and AgCl would migrate to the CB of AgIO_3_. However, the CB potentials of AgIO_3_ is excessively positive contrast to O_2_/•O^2−^ (−0.046 eV vs. NHE), therefore it cannot reduce O_2_ to produce •O^2−^. While the •O^2−^ radicals have been detected during the trapping experiments, suggesting the Fermi energy of the AgIO_3_ would move more negative position in the photocatalytic system to match well with the energy level of its surrounding medium^18,36^. Meanwhile, the whole energy level of the AgIO_3_ rearranged and the conduction band of the AgIO_3_ became more negative. Hence, it is possible to reduce O_2_ to produce •O^2−^ by the photoinduced electrons on the CB of AgIO_3_ in this photooxidative process. At the same time, the photogenerated h^+^ was remained and accumulated on the VB of AgI (2.37 eV) because that it is more negative than the VB of AgIO_3_ (4.01 eV) and AgCl (3.19 eV), which could not oxidize -OH into •OH with the oxidative potential of 2.6 V vs. NHE^37,38^. The above mentioned results are in accordance with the trapping experiment. While under the irradiation of visible light (Fig. 8d), only AgI can be excited because of the native narrow band gap (E_g_ = 2.8 eV). The photogenerated electrons will transfer from AgI to AgIO_3_ and reduce O_2_ to produce •O^2−^, meanwhile the photogenerated holes which remained on the VB of AgI will oxidize MO directly owing to its strong oxidative capacities. Consequently, no matter what the excitation light source used, the separation of photogenerated electron-hole pairs could be significantly improved because of the synergistic effect among the ternary materials. Besides, the conversion of a part of I^5+^ to I^−^ during the photocatalytic process may also have effects on the oxidation of dyes^39^, as seen in Supplementary Fig. S7. Meanwhile, the generated silver atomic clusters during photocatalytic process can absorb visible light and thus induce the appearance of photogenerate electrons and holes, which is also benefit for enhancing the visible light photocatalytic performance of composite materials (Supplementary Fig. S8).

## Conclusion

In conclusion, the novel AgCl/AgI/AgIO_3_ composites were designed and synthesized by a facile and rapid one-pot *in situ* solid-state chemical technique. The AgCl/AgI/AgIO_3_ composites displayed greatly upgraded photocatalytic performance for the degradation of MO under both UV light and visible light irradiation compared with pure AgIO_3_, AgCl, AgI, and AgI/AgIO_3_, which mainly depends on the enhanced light adsorption in full spectrum and the efficient charge separation on the surface. The efficient charge separation of the AgCl/AgI/AgIO_3_ composites is attributed to the photogenerated electrons of AgI or AgCl could eventually transfer to the CB of AgIO_3_, the holes would migrate to the VB of AgI simultaneously. This work may offer a simple avenue to realize rationally design of Ag-based heterojunction structures with tunable photocatatlytic performance.

## Experiment

### Preparation of AgCl/AgI/AgIO_3_ composites

Every chemical used in this article is of analytical pure and used without further purification. The AgCl/AgI/AgIO_3_ composites were synthesized by a room-temperature one-pot *in situ* solid-state reducing reaction. Firstly, 5 mmol of AgNO_3_ was weighted and grinded into fine powder, then 5 mmol of KIO_3_ was added and grinded for about 10 min, finally, a certain amount of HONH_3_Cl was added into the above mixture and ground for 30 min to ensure the completeness of the reaction. After reaction, the precipitate of the samples was collected and washed thoroughly with distilled water and absolute ethanol, and dried at room temperature in air for 1 h. The as-prepared composites with the amount of HONH_3_Cl of 1 mmol, 2 mmol, 3 mmol and 4 mmol are marked as AgCl/AgI/AgIO_3_-1, AgCl/AgI/AgIO_3_-2, AgCl/AgI/AgIO_3_-3 and AgCl/AgI/AgIO_3_-4, respectively.

The preparation process of AgIO_3_ was the same as the preparation of AgCl/AgI/AgIO_3_ composites except for the absence of HONH_3_Cl.

### Characterization

X-ray diffraction (XRD) was served to analyze the crystalline phase by a Bruker D8 using filtered Cu Kα radiation (1.54056 Å) with an operating voltage of 40 kV and a beam current of 40 mA. Energy dispersive X-ray spectrometer (EDX) and mapping was obtained on an Oxford 2000 with the accelerating voltage of 200 kV. Raman spectrum was measured using a Bruker spectrometer with a solid-state laser (excitation at 532 nm, 10 mW) at room temperature in the range of 1000-40 cm^−1^. The X-Ray Photoelectron Spectroscopy (XPS) (ESCALAB 250Xi, Thermo Fisher Scientific, USA) was applied to investigate the chemical composition and the chemical states employing Al Kα 1486.6 eV. Field emission scanning electronic microscopy (FESEM, Hitachi, SU8010 at 5 kV) was performed to obtain the morphology of the samples. Transmission electron microscope (TEM) was acquired on a JEM-2100F at 200 kV (JEOL, Japan). The UV-vis diffuse reflectance spectroscopy was conducted on Hitachi U-3900H Spectrophotometer, using BaSO_4_ as the reference, which was employed to measure the intrinsic absorption wavelength of the catalyst. Photoluminescence (PL) spectra of the as-prepared samples were measured on a fluorescence spectrophotometer (F-4500, Hitachi, Japan). The UV-Vis absorption spectrum was conducted on Hitachi U-3010 Spectrophotometer, which was used to analysis the absorption spectra of the degraded dyes. The photocatalytic experiments were carried out in an XPA-1 photochemical reactor (Xujiang Electromechanical Plant, Nanjing, China).

### Photocatalytic activity test

The photocatalytic activity of the as-prepared samples were systematically evaluated by the degradation of MO under UV light (300 W Hg lamp, 6.06 mW/cm^2^) illumination for 12 min or visible light (350 W Xe lamp with a ≥420 nm cutoff optical filter, 1.9 mW/cm^2^) irradiation for 50 min, respectively. Both of the two lamps have been bought with XPA-1 photochemical reactor (Xujiang Electromechanical Plant, Nanjing, China). 25 mg of AgCl/AgI/AgIO_3_ powders was ultrasonically dispersed in 50 ml MO solution (10 mg/L for UV light, 5 mg/L for visible light). Prior to illumination, the suspension was magnetically stirred in the dark for 0.5 h to reach the adsorption-desorption equilibrium between MO and the catalysts. During the whole process including adsorption in dark and UV light irradiation, 3 mL of the suspension was collected and centrifuged to remove the photocatalyst particles, and then analyzed by recording the UV-vis spectrum of MO at 463 nm within a given time intervals. The rhdamine B (RhB, 5 mg/L), methylene blue (MB, 5 mg/L), phenol (10 mg/L), bisphenol A (BPA, 10 mg/L) and tetracycline hydrochloride (10 mg/L) were also employed to evaluate the photocatalytic activity of the AgCl/AgI/AgIO_3_-3 composite. Besides, the wavelength dependent photocatalytic activity over the AgCl/AgI/AgIO_3_-3 composite was evaluated by the 300 W Xe lamp with different band-pass filter (CEL-SPH2N, Beijing, China), the corresponding optical power intensity was displayed in Supplementary Fig. S6.

### Photoelectrochemical measurements

An electrochemical analyzer (CHI 660E) with a standard three electrode configuration was served to measure the photocurrents and electrochemical impedance spectroscopy (EIS) of the samples at room temperature. The photocurrent intensity was measured under intermittent illumination with a 300 W Xe lamp at 0.3 V. 0.5 mol·L^−1^ Na_2_SO_4_ aqueous solution was used as the electrolyte, the saturated Ag/AgCl electrode served as reference electrode, the Pt wire employed as counter electrode and the as-prepared samples coated on F-doped SnO_2_-coated glass (FTO glass) substrate used as working electrode (photoanode). To fabricate the photoanode, 5 mg the as-prepared powder was ultrasonic disperse in 1 ml ethanol homogeneously, then the mixture were spread on an F-doped SnO_2_-coated glass (FTO) substrate (1.0 cm × 2.0 cm), finally dried at 100 °C in an oven.

## Electronic supplementary material

Supporting Information
