# Supplementary material for: In situ solid-state fabrication of hybrid AgCl/AgI/AgIO3 with improved UV-to-visible photocatalytic performance
Source: Sci Rep. 2017 Sep 28;7:12365. doi: 10.1038/s41598-017-12625-8 (PMC5620062; doi:10.1038/s41598-017-12625-8)
Supplement: Supplementary file 1 — Supporting Information [file 41598_2017_12625_MOESM1_ESM.pdf]

In situ solid-state fabrication of hybrid $AgCl/AgI/AgIO_3$ with improved UV-to-visible photocatalytic performance

Jing Xie, Yali Cao*, Dianzeng Jia*, Yizhao Li, Kun Wang, Hui Xu

Key Laboratory of Energy Materials Chemistry, Ministry of Education, Key Laboratory of Advanced Functional Materials, Autonomous Region, Institute of Applied Chemistry, Xinjiang University, Urumqi, 830046 Xinjiang, P. R. China.

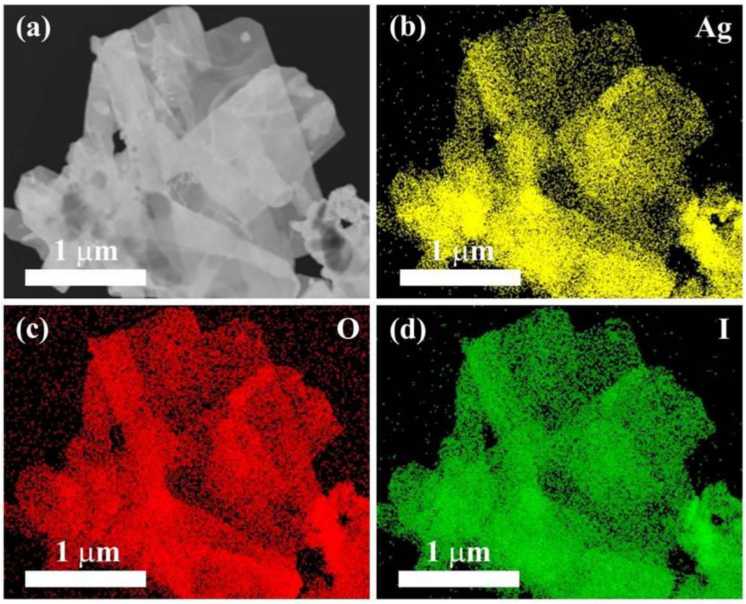

Supplementary Fig. S1. EDS mapping of pure $AgIO_3$.

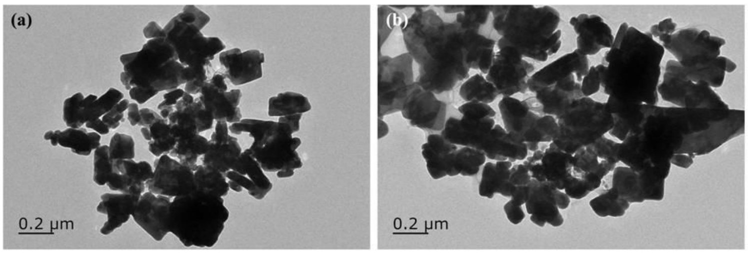

Supplementary Fig. S2. The low-magnification TEM of $AgCl/AgI/AgIO_3$-3.

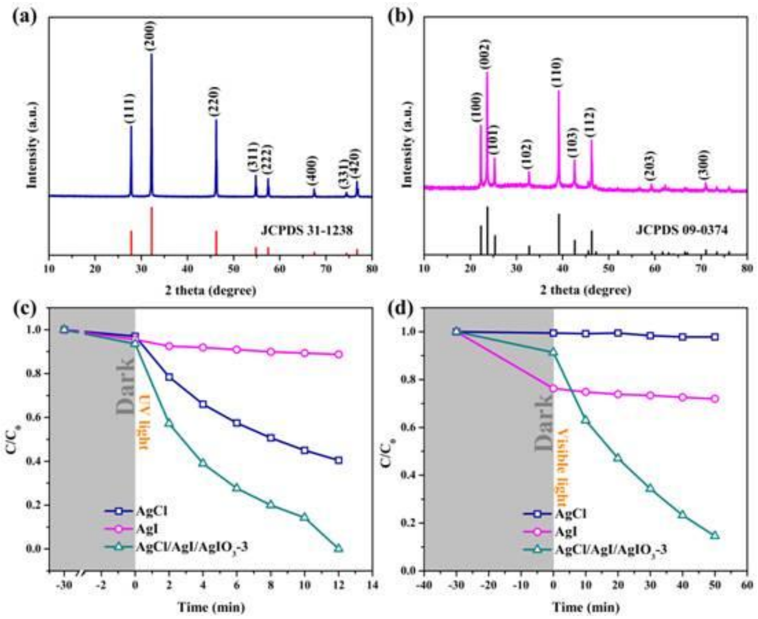

Supplementary Fig. S3. The XRD patterns of pure AgCl (a) and AgI (b), Degradation efficiencies of MO over pure AgCl, AgI and AgCl/AgI/$AgIO_3$-3 composites under UV light (c) or visible light irradiation (d).

The preparing methods of pure AgCl and AgI are largely the same as the fabrication of $AgIO_3$. The raw materials for preparing pure AgCl and AgI are $AgNO_3$ and $HONH_3Cl$, $AgNO_3$ and NaI, respectively, and the molar quantities of them are all 5 mmol. Fig. S3 shows the XRD patterns of the as-prepared pure AgCl and AgI, which is match well with cubic AgCl (JCPDS 31-1238) and AgI (JCPDS 09-0374), respectively. This indicates that the pure AgCl and AgI can also be obtained by the facile room-temperature solid-state chemical reaction.

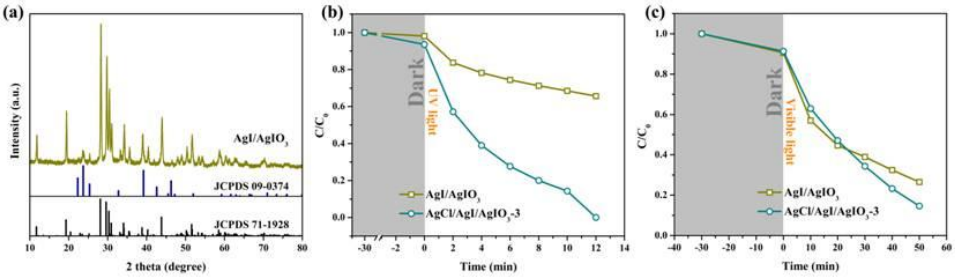

Supplementary Fig. S4. The XRD patterns of AgI/$AgIO_3$ composite (a), Degradation efficiencies of MO over AgI/$AgIO_3$ and AgCl/AgI/$AgIO_3$-3 composite under UV light (b) or visible light irradiation (c).

The preparing method of AgI/$AgIO_3$ is largely the same as the fabrication of

AgCl/AgI/$AgIO_3$ composite except for the substitute of the reductant, and $(NH_2OH)_2 \cdot H_2SO_4$ was used as the reductant in the preparation of AgI/$AgIO_3$ composite. Fig. S4 shows the XRD pattern of the as-prepared AgI/$AgIO_3$ composite, all the diffraction peaks can be ascribed to cubic orthorhombic $AgIO_3$ (JCPDS 71-1928) and AgI (JCPDS 09-0374), respectively. This indicates that the AgI/$AgIO_3$ composite can also be obtained via the room-temperature solid-state chemical reaction.

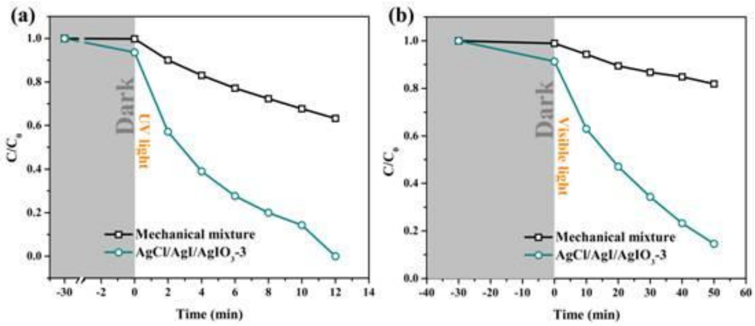

Supplementary Fig. S5. The degradation efficiencies of MO over the mechanical mixtures of AgCl, AgI, $AgIO_3$ and the AgCl/AgI/$AgIO_3$-3 composites was contrasted under UV light (a) or visible light irradiation (b).

Compared with the *in situ* reactive generated AgCl/AgI/$AgIO_3$-3 composites, the degradation efficiencies of the mechanical mixtures is far below than the AgCl/AgI/$AgIO_3$-3 composites, indicating the importance of *in situ* reaction.

Supplementary Table S1. The optical power, reaction rate and apparent quantum yield over the AgCl/AgI/$AgIO_3$-3 composite under the 300 W Xe lamp with different band-pass filter.

Wavelength (nm)	365	380	400	420	450	475	500	550	600	650
Optical power (mW cm^{-2})	177.0	153.0	46.0	45.0	54.4	59.2	56.5	59.2	62.1	72.8
Reaction rate $\times 10^{-8}$ (mol s^{-1})	1.92	1.65	3.78	3.72	1.14	0.99	1.19	1.22	1.72	1.36

The incident optical power of the light sources has been measured by an optical power meter (CEL-NP2000). The photocatalytic activity of the as-prepared samples were systematically evaluated by the degradation of MO under UV light (300 W Hg lamp, 6.06 mW/cm^2) illumination for 12 min or visible light (350 W Xe lamp with a ⩾420 nm cutoff optical filter, 1.9 mW/cm^2) irradiation for 50 min, respectively. Both of the two lamps have been bought with XPA-1 photochemical reactor (Xujiang Electromechanical Plant, Nanjing, China). Besides, A 300 W Xe lamp with a different optical reflector (VISREF: 350-780 nm) or filters (QD365: 365 ±15 nm, QD380: 380 ±15 nm, QD400: 400 ±15 nm, QD420: 420 ±15 nm, QD450: 450 ± 15 nm, QD475: 475 ± 15 nm, QD500: 500 ± 15 nm, QD550: 550 ± 15 nm, QD600: 600 ± 15 nm, QD650: 650 ± 15 nm) was employed as the light sources, which was bought from CE-AULIGHT Technology Co., Ltd (CEL-SPH2N, Beijing, China). The optical power of the 300 W Xe lamp with different filters were also measured and displayed in Table S1.

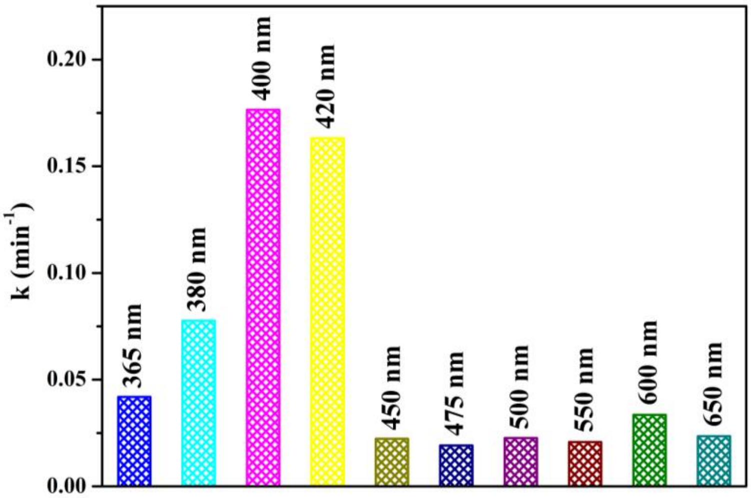

Supplementary Fig. S6. The photocatalytic activity of AgCl/AgI/$AgIO_3$-3 composite depended on wavelength.

The apparent quantum yield over the AgCl/AgI/$AgIO_3$-3 composite under the 300 W Xe lamp with different band-pass filter was calculated by the follows[1-3]:

$E = hc/\lambda$ (1)

$E' = AE$ (2)

$F = P/E'$ (3)

$\Phi = r/(F\times a)$ (4)

where E (J), λ (m), E' (J mol^{-1}), P (W cm^{-2}), F (mol s^{-1} cm^{-2}), r (mol s^{-1}), a (cm^{2}) and Φ are the energy of a photon, wavelength, energy of mole of photons, optical power intensity, photon flux intensity, reaction rate, irradiation area and apparent quantum yield, respectively. While h, c and A are the planck constant, speed of light and avogardo constant, respectively. The values of these constants (h, c and A) are 6.62607× 10^{-34} J s, 2.99792458 × 10^{8} m s^{-1}, 6.02214086 × 10^{23} mol^{-1}, respectively. The calculated apparent quantum yields are displayed in Fig. S6, which is almost consistent with the wavelength dependent photocatalytic activitiy.

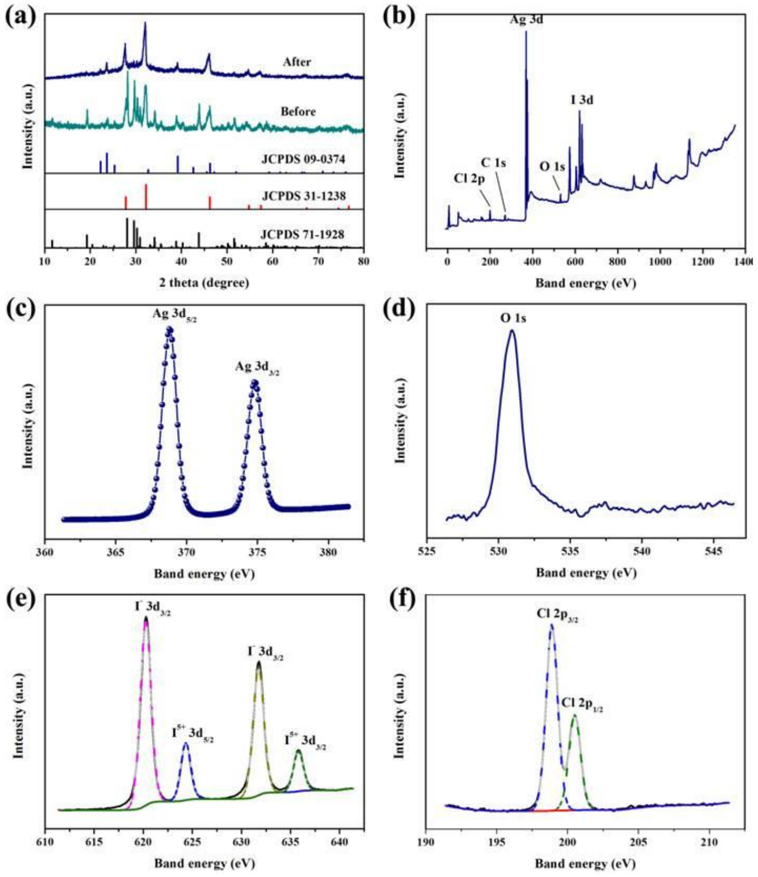

Supplementary Fig. S7. XRD and XPS of the after photocatalysis for $AgCl/AgI/AgIO_3$-3 composite.

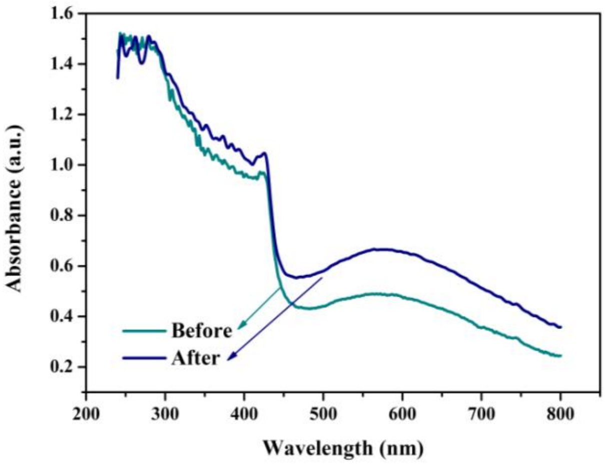

Supplementary Fig. S8. UV-vis diffuse reflectance spectroscopy of the $AgCl/AgI/AgIO_3$-3 composite.

As seen in the Fig. S7a., with the proceeding of recycling experiments, the characteristic peaks of $AgIO_3$ decreased in intensity gradually, while the characteristic peaks of AgI increased in intensity simultaneously. In addition, the corresponding high-resolution XPS spectra in Fig. S7e. displayed that two peaks at 624.08 eV and 635.58 eV which can be ascribed to I^{5+} $3d_{5/2}$ and I^{5+} $3d_{3/2}$, and meantime the peaks at 619.98 eV and 631.48 eV match the features of I^- $3d_{5/2}$ and I^- $3d_{3/2}$, respectively. Compared with $AgCl/AgI/AgIO_3$-3 composite before the photocatalytic reaction (Fig. 2c), the relative content of I^- increased and the content of I^{5+} decreased obviously, indicating the conversion of the I^{5+} to I^- during the photocatalytic process. Hence, there is a little decrease after three consecutive cycles, which could attribute to the conversion of a part of I^{5+} to I^-. The reaction changed the relative contents of AgCl, AgI and $AgIO_3$, which is the disadvantage for recycling. In addition, the enhanced photooxidative abitlity of $AgCl/AgI/AgIO_3$-3 photocatalyst would be attributed to the conversion of a part of I^{5+} to I^- which also can oxidize dyes and the synergistic effect among the ternary materials. To further confirm the composition of the used $AgCl/AgI/AgIO_3$-3 composite, UV-vis diffuse reflectance spectroscopy techniques were also employed to analyze the fresh and used sample. As seen in Fig. S8., the visible light absorption ability of the used catalyst was much better than that of the fresh, illustrating that the emergence of the silver atomic clusters due to the silver is in favor of light absorption. The generated silver atomic clusters can absorb visible light

and thus induce the appearance of photogenerate electrons and holes owing to the dipolar character and SPR of metallic Ag, which is also benefit for enhancing the visible light photocatalytic performance of composite materials.

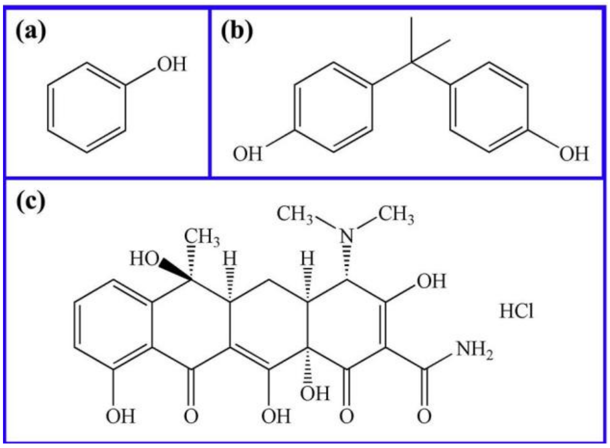

Supplementary Fig. S9. The structural formulas of phenol (a), bisphenol A (b) and tetracycline hydrochloride (c).

In this article, three phenolic compounds (phenol, bisphenol A and tetracycline hydrochloride) were selected to evaluate the photocatalytic activity of the hybrid $AgCl/AgI/AgIO_3$ and their structural formulas were displayed in Fig. S9. The structures of them are similar and they all have benzene-ring. It is well known that benzene-ring is very stable due to its π-conjugated systems, which means the ring-opening reaction is difficult to occur. Therefore, as for bisphenol A and tetracycline hydrochloride, the C=O bond, C-N bond, C-O bond and C-C bond that does not belong to the benzene-ring would be broken during the photocatalytic process. It is very difficult to undergo further degradation until only benzene-ring remains. Besides, the degradation rate of bisphenol A and tetracycline hydrochloride decreased gradually, which is consistent with the analysis of structure. While for phenol, primarily, the P-π conjugate that exist in phenol makes the C-O bond very firm, leading to the fracture of C-O bond is very difficult to occur in phenol. Besides, even if the C-O bond was broken, the ring-opening reaction of benzene is also hard to happen. Thus, the composite does not show an excellent degradation effects on phenol.

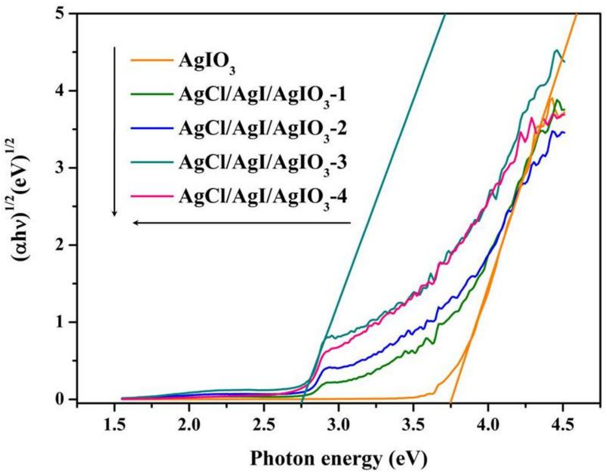

Supplementary Fig. S10. The $(\alpha h\gamma)^{1/2}$ versus photon energy (*hv*) curve of pure $AgIO_3$ and $AgCl/AgI/AgIO_3$ heterostructures.

The band gap energy of as-prepared samples can be calculated by the equation: $\alpha h\gamma = A(h\gamma\text{-}E_g)^{n/2}$, where α, γ, E_g, and A are the absorption coefficient, light frequency, band gap, and constant, respectively. The value of n is 4 for pure $AgIO_3$ and $AgCl/AgI/AgIO_3$ heterostructures, indicating they are the indirect band gap materials [31]. In Fig. S10, the Eg value was calculated through the Tauc plot extrapolation based on the spectral response. The evaluated band gap value is 3.8 eV, corresponding to pure $AgIO_3$. In addition, we can see that the absorption edge of the $AgCl/AgI/AgIO_3$ heterostructures firstly shifts to narrow band gap and then increase as the amount of $HONH_3Cl$ increases, the $AgCl/AgI/AgIO_3$-3 heterostructures has the smallest band gap and the value is 2.8 eV. These results suggest that all the $AgCl/AgI/AgIO_3$ heterostructures samples have photoabsorption abilities in visible light region and thus can be used as potential visible light photocatalysts. Furthermore, the potentials of the valence band (VB) and conduction band (CB) of semiconductors can be calculated with the two formulas proposed by Butler and Ginley: $E_{VB} = \chi - E_e + 0.5\ E_g$, $E_{CB} = E_{VB} - E_g$, where E_{VB}, E_{CB} and E_g are the VB potential, CB potential and band gap of the as-prepared material, respectively; E_e is the energy of free electrons on the hydrogen scale (ca. 4.5 eV vs NHE); χ is the absolute electronegativity of the corresponding semiconductor material. Based on the above formulas and the optical absorption spectrum, the VB potential and the CB potential was displayed as Table S1.

Supplementary Table S2. The calculated χ, CB and VB potentials of AgCl, AgI and $AgIO_3$.

	χ	E_g (eV)	E_{VB} (eV)	E_{CB} (eV)
**AgCl**	6.07	3.3	3.19	-0.06
**AgI**	5.47	2.8	2.37	-0.42
**$AgIO_3$**	6.63	3.8	4.01	0.26

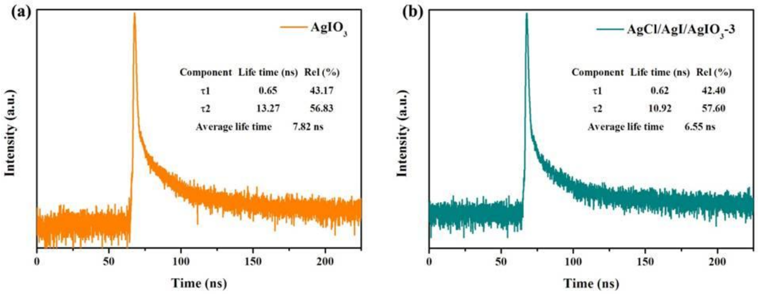

Supplementary Fig. S11. Time resolved photoluminescence spectra of pure $AgIO_3$ (a) and AgCl/AgI/$AgIO_3$-3 composite (b).

Time resolved photoluminescence spectra of pure $AgIO_3$ and AgCl/AgI/$AgIO_3$-3 composite were studied. As presented in Fig. S11., the average lifetimes and their percent contributions were determined from a double exponential fitting. By analysis, both the shorter lifetime (τ1) and longer lifetime (τ2) of the AgCl/AgI/$AgIO_3$-3 composite decreased as compared to pure $AgIO_3$. Besides, the contribution of shorter lifetime decreased from 43.17% to 42.40%, and that of the longer lifetime increased from 56.83% to 57.6%. The shorter lifetime of intrinsic fluorescence is associated with the radiative decay from excited state to the ground state. The decreased contribution of shorter lifetime means that lower quantity of quickly recombined charge carriers. The decreased average lifetime and changed contributions indicate more non-radiative transfer of photogenerated charge carriers in AgCl/AgI/$AgIO_3$-3 composite, which is induced by synergistic effects and provided better charge transfer pathways[4,5].

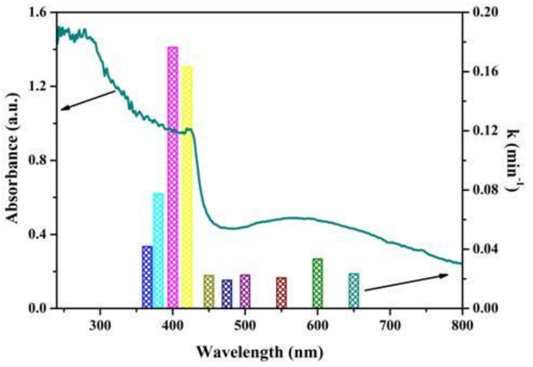

Supplementary Fig. S12. The UV-Vis diffuses reflectance spectra and the wavelength-dependent photocatalytic activities of $AgCl/AgI/AgIO_3$-3.

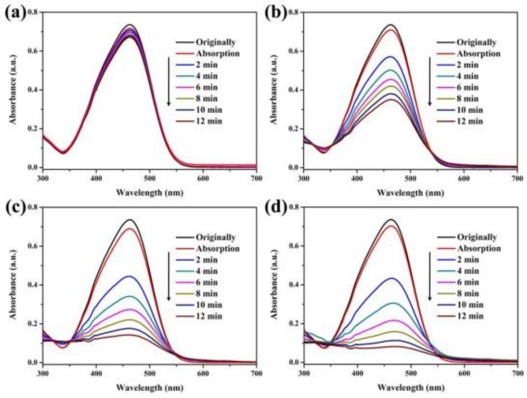

Supplementary Fig. S13. The temporal adsorption spectra of MO in the presence of pure $AgIO_3$-1 (a), $AgCl/AgI/AgIO_3$-1 (b), $AgCl/AgI/AgIO_3$-2 (c) and $AgCl/AgI/AgIO_3$-4 (d) during the photodegradation under UV light irritation.

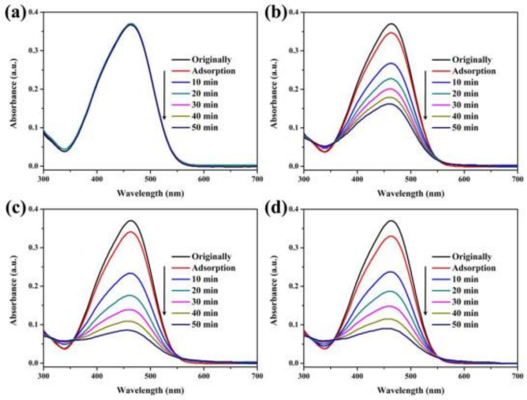

Supplementary Fig. S14. The temporal adsorption spectra of MO in the presence of pure $AgIO_3$-1 (a), $AgCl/AgI/AgIO_3$-1 (b), $AgCl/AgI/AgIO_3$-2 (c) and $AgCl/AgI/AgIO_3$-4 (d) during the photodegradation under visible light irradiation ($\lambda \geqslant 420$ nm).

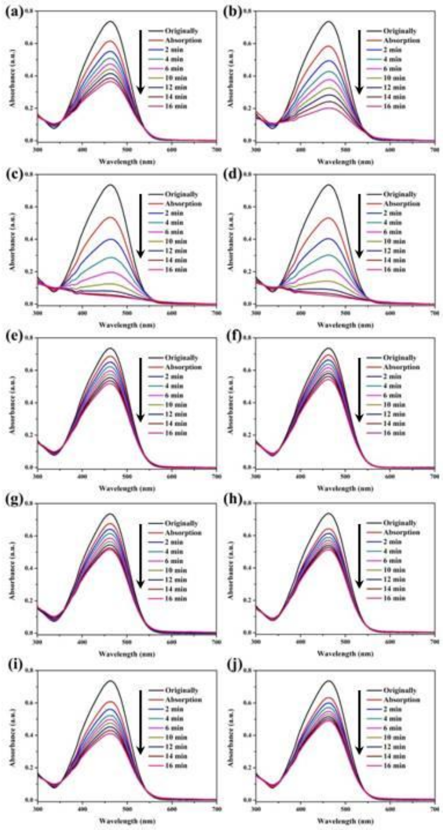

Supplementary Fig. S15. The temporal adsorption spectra of MO in the presence of AgCl/AgI/$AgIO_3$-3 during the photodegradation depended on different wavelength (a) 365 nm, (b) 380 nm, (c) 400 nm, (d) 420 nm, (e) 450 nm, (f) 475 nm, (g) 500 nm, (h) 550 nm, (i) 600 nm and (j) 650 nm.

References

1. Ohtani, B. Revisiting the fundamental physical chemistry in heterogeneous photocatalysis: its thermodynamics and kinetics, Phys. Chem. Chem. Phys. **16**, 1788-1797 (2014).

2. Muñoz-Batista, M.J., Kubacka, A., Hungría, A.B. & Fernández-García, M. Heterogeneous photocatalysis: Light-matter interaction and chemical effects in quantum efficiency calculations, J. Catal. **330**, 154-166 (2015).

3. Kisch, H. Semiconductor photocatalysis, Chapter 2, Molecular photochemistry Page 9-25, Nachrichten Aus Der Chemie **50**, 1078-1082 (2014).

4. Fu, J.W. et al. Hierarchical porous O-doped g-C_3N_4 with enhanced photocatalytic CO_2 reduction activity, Small **1603938**, 1-9 (2017).

5. Dozzi, M.V., Andrea, C.D., Ohtani, B., Valentini, G. & Selli, E. Fluorine-doped

TiO_2 materials: photocatalytic activity vs time-resolved photoluminescence, J. Phys. Chem. C **117**, 25586-25595 (2013).
